# The expanding Asgard archaea and their elusive relationships with Eukarya

**DOI:** 10.1002/mlf2.12012

**Published:** 2022-03-24

**Authors:** Violette Da Cunha, Morgan Gaïa, Patrick Forterre

**Affiliations:** ^1^ CEA, CNRS, Institute for Integrative Biology of the Cell (I2BC) Université Paris‐Saclay Gif‐sur‐Yvette France; ^2^ Génomique Métabolique, Génoscope, Institut François Jacob, CEA, CNRS Univ. Evry, Université Paris‐Saclay Evry France; ^3^ Département de Microbiologie, Institut Pasteur Paris France

**Keywords:** Asgard, horizontal gene transfer, molecular phylogeny, tree of life

## Abstract

The discovery of Asgard archaea and the exploration of their diversity over the last 6 years have deeply impacted the scientific community working on eukaryogenesis, rejuvenating an intense debate on the topology of the universal tree of life (uTol). Here, we discuss how this debate is impacted by two recent publications that expand the number of Asgard lineages and eukaryotic signature proteins (ESPs). We discuss some of the main difficulties that can impair the phylogenetic reconstructions of the uTol and suggest that the debate about its topology is not settled. We notably hypothesize the existence of horizontal gene transfers between ancestral Asgards and proto‐eukaryotes that could result in the observed abnormal behaviors of some Asgard ESPs and universal marker proteins. This hypothesis is relevant regardless of the scenario considered regarding eukaryogenesis. It implies that the Asgards were already diversified before the last eukaryotic common ancestor and shared the same biotopes with proto‐eukaryotes. We suggest that some Asgards might be still living in symbiosis today with modern Eukarya.

## INTRODUCTION

**Figure 1:**
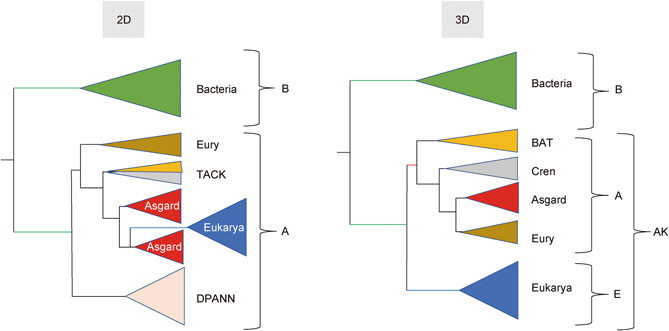
Two vs. three domains models for the universal tree of life. In the two domains (2D) model, Eukarya (E) branch within Archaea (A) and should be formally considered to be Archaea themselves (otherwise Archaea being paraphyletic are not a valid taxon). This schematic tree is based on one possible result of Liu et al.^13^ in 2021, in which eukaryotes belong to the Asgard superphylum. In the three domains, model Archaea (A) and Eukarya (E) form a clade dubbed Arkarya (AK) in Forterre^8^. The bacterial branch (green) is much longer than the eukaryotic and archaeal branches (blue and red, respectively). The archaeal branch (red) is very short and only present in the three domains (3D) model. This schematic tree is based on Da Cunha et al.^5^. Eury(eury): Euryarchaeota; Cren: Crenarchaeota. TACK is the acronym for the clade grouping Thaumarchaea, Aigarchaea, Crenarchaea, and Korarchaea; DPANN is the acronym for the clade grouping Diapherotrites, Parvarchaea, Aenigmarchaea, Nanohaloarchaea, and Nanoarchaea; BAT is the acronym for the clade grouping Bathyarchaea, Aigarchaea, and Thaumarchaea^9^. The trees are rooted in the bacterial branch, based on comparative analysis of ribosomal protein distribution^8^.

The discovery of Asgard archaea has revitalized an intense debate about the universal tree of life (uTol) topology^1^, ^2^. Proponents of the two primary domain (2D) uTol propose a scenario in which Eukarya emerged within Archaea, with Eukarya being a subgroup of the Asgard superphylum^3^, ^4^, whereas others still support a uTol in which all three domains (3D) are monophyletic, with Asgards nested within Archaea^5^, ^6^, ^7^ (Figure 1).

**Figure 2:**
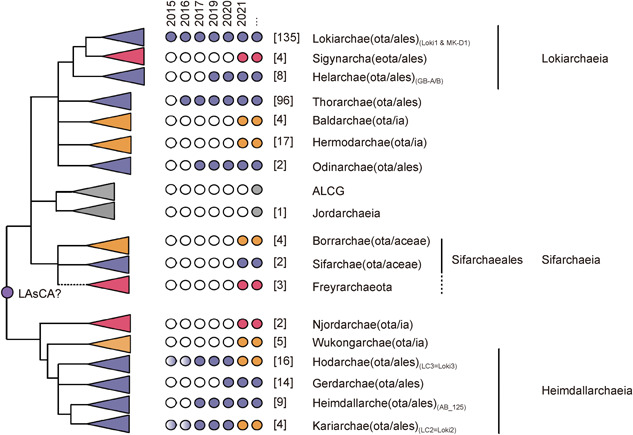
Schematic representation of the known diversity of the Asgard archaea including the recently published lineages. In this schema, we indicate in orange and red the newly discovered lineages introduced in the two publications of Liu et al.^13^ and Xie et al.^14^, respectively, and in grey new lineages not present in these publications corresponding to the Sifarchaea^12^, the Jordarchaeia^16^ and Asgard Lake Cootharaba^16^. The schema was designed combining the trees of all these publications. The suffix within brackets indicates that, depending on the authors, these lineages are considered to be Phylum (ota), Families (aceae), or Orders (ales). We locate the position of LAsCA (last Asgard archaeal common ancestor) based on the root observed in most phylogenetic trees, and used by Liu et al. to discuss the ESPs distribution. For each Asgard lineage, we also indicate the year of publication of the genome of its first representative, and changes in taxonomy are indicated by color changes of the dots. The light purple dots indicate that Asgards of the Karia and Hodar and lineages were described before 2017 as Loki 2 and Loki 3, respectively^1^. In addition, the number of genomes available for each lineage is indicated within brackets.

The number of proposed Asgard phyla has continuously increased in recent years, from 1 in 2015 (Lokiarchaeota) to 18 in late 2021 (Figure 2)^1^, ^2^, ^10^, ^11^, ^12^, ^13^, ^14^, ^15^, ^16^. In a recent study, Liu et al.^13^ described six new Asgard phyla from an analysis of 75 metagenome‐assembled genomes (MAGs), while three additional phyla were described by Xie et al.^14^ from an analysis of 128 new MAGs. The characterization of these new Asgard lineages led to the identification of several new eukaryotic signature proteins (ESPs) but also emphasizes their patchy distribution among the various Asgard lineages.

Liu and colleagues obtained a 2D uTol, with Eukarya most likely branching within Asgards as a sister group to an extended clade grouping the Wukong lineages with lineages previously considered to be Heimdallarchaeota^13^. Their phylogenetic analysis was based on a concatenation of 29 universal proteins that only partially overlap with the lists of universal markers previously used^1^, ^2^, ^4^, ^17^. Xie and colleagues also obtained a 2D uTol but with Eukarya as sister group to the Njord lineage (a close relative of Wukong and Heimdall); their analysis was based on 21 universal proteins previously used by Williams et al.^4^, who also obtained a 2D tree.

Although both teams concluded in favor of a 2D tree, Liu et al.^13^ cautiously mentioned that “further phylogenomic study with an even broader representation of diverse archaeal lineages, extended sets of phylogenetic markers and—possibly—more sophisticated evolutionary models are required to clarify the relationships between Archaea and eukaryotes”.

We discuss here these assumptions in light of our previous results and new data that have suggested various biases that could favor 2D topologies. We also propose that some Asgard ESPs and universal proteins could have been recruited from proto‐eukaryotes, possibly explaining the patchy distribution of ESPs and the atypical placement of some Asgard lineages in single‐gene uTols.

Rinke and colleagues recently proposed to consider the whole Asgard clade as a single phylum and the various Asgard lineages either as families or orders, with some of them being grouped in classes^16^. Since it is still an ongoing debate at the time of writing this review^18^, we will here use the more neutral term “lineage” for the various subgroups of Asgards (e.g., Heimdall lineage for Heimdallarchaeota/ales).

## BROADER REPRESENTATION OF ARCHAEAL LINEAGES CAN FAVOR 2D MODELS

Compared to previous studies, Liu, Xie, and their respective colleagues used an expanded species data set, especially enriched in Archaea and Asgards. This can be problematic since simulation experiments have shown that overrepresentation of Archaea in uTols can favor 2D models^19^. This could be due to the very short internal branch defining the archaeal monophyly in the 3D uTol (in red in Figure 1) since internal branches become progressively shorter as taxonomic sampling increases^20^. Overbalanced representation of diverse archaeal lineages could thus introduce a bias if the representation of Bacteria and Eukarya is not increased accordingly^19^. In a recent reconstruction of phylogenetic trees, including Archaea and Bacteria, the authors noted that removal of some genomes can improve the phylogenetic inference, and that larger sets do not necessarily improve phylogenetic accuracy^21^. They noticed that oversampling of some groups relative to others can influence the placement of other groups in the tree.

It is also important to keep in mind that phylogenetic trees are the final output of successive analytical steps. Among them, multiple sequence alignments are critical, and despite undeniable improvements in their methods, the risk of misalignment increases along with the size of the data set. Misalignments of universal proteins due to domain inversion/substitution or mismatch have been detected by Nasir et al.^7^ in 42% of the universal markers used in the first Asgard paper. Since some of these markers are also present in the data sets of Liu et al. and of Xie et al. (reference^22^ and personal observation), it will be important to check the new alignments for possible mistakes. Notably, the risk of misalignment is increased in the case of taxon oversampling and with the inclusion of fast‐evolving species, two criteria frequently observed in uTol studies^7^, ^23^.

## THE CONUNDRUM OF FAST‐EVOLVING SPECIES

**Figure 3:**
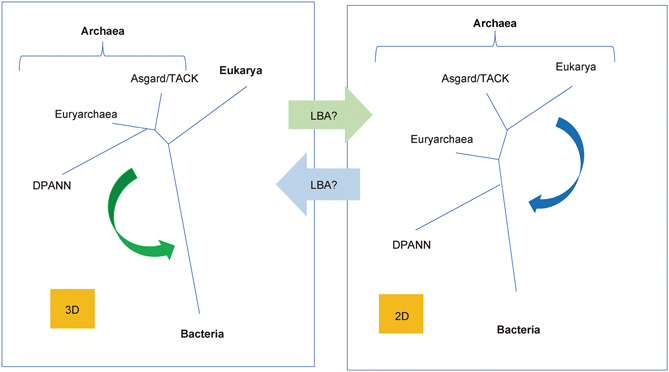
Two possible long branch attraction effects that could transform a 3D uTol into a 2D uTol and vice versa. On the left, the green arrow suggests that the attraction of DPANN and Euryarchaeota by Bacteria could transform the correct 3D tree into the 2D tree on the right. On the right, the blue arrow suggests that the attraction of eukaryotes by Bacteria could transform the correct 2D tree into the 3D tree on the left.

A major feature of the uTol is the difference between the lengths of its different stem branches (Figure 1). The topology of the tree is therefore very sensitive to long branch attraction (LBA) artifacts. Simulation experiments have shown that even the best models cannot cope with LBA when the branch of the outgroup is especially long^24^, which is the case for the bacterial branch in uTols (green in Figure 1)^5^, ^25^, ^26^. The presence of many fast‐evolving Archaea, such as the Diapherotrites, Parvarchaea, Aenigmarchaea, Nanohaloarchaea, Nanoarchaea (DPANN) and *Methanopyrus kandleri* in the data set of Liu et al., can thus be problematic. We have previously shown that removing fast‐evolving Archaea from the species data set of the first Asgard paper dramatically increased the number of single‐protein uTols with 3D topology^5^. [Correction added on June 6, 2022, after first online publication: A citation for reference 17 has been deleted from this sentence.] The presence of fast‐evolving Archaea could probably favor a 2D topology through their attraction by the long branch of Bacteria (Figure 3). Considering the possible close evolutionary relationships between some DPANN and Euryarchaeota^27^, this could result in the branching of Eukarya within Archaea, transforming a 3D uTol into a 2D uTol (Figure 3).

**Figure 4:**
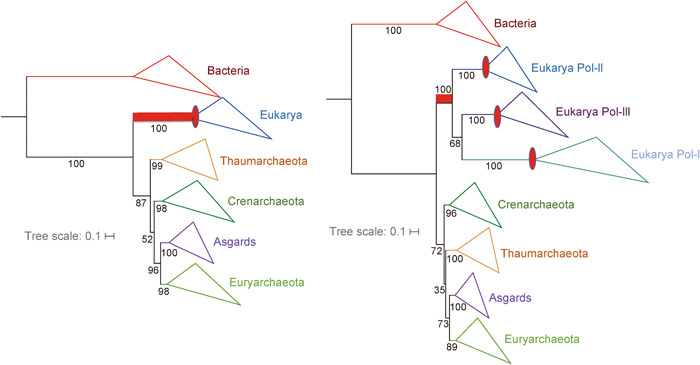
RNA polymerase tree remains 3D after shortening the eukaryal branch. Maximum‐likelihood (ML) phylogenetic trees were constructed based on the concatenated two largest DNA‐dependent RNA polymerase subunits. ML phylogenetic trees are of the Bacteria, eukaryotes, and Archaea, including Asgard sequences from Lokiarchaeota (1 genome), Heimdallarchaeota (2 genomes), Odinarchaeota (1 genome), and Thorarchaeota (3 genomes). The tree on the left is from data set^5^, while the tree on the right corresponds to the same data set and parameters but after the inclusion of sequences of the eukaryotic RNA polymerases I and III from data set^28^. For consistency, the same method has been applied to both and is described in Da Cunha et al.^5^. Briefly, the sequences were aligned with MAFFT v7 (default settings^29^) and trimmed with BMGE (BLOSUM30 matrix^30^). PhyML v3^31^ was used for tree reconstruction with the BEST option for the tree search topology operations after the model was chosen according to the Akaike information criterion from ProtTest v3^32^. The scale bars represent the average number of substitutions per site. Supports at branch correspond to nonparametric bootstrap (out of 100).

It is likely that solving the uTol topology is a difficult exercise because of a complex interplay of branch attraction effects between Bacteria, Euryarchaeota, Asgards, and Eukarya. This highlights the importance of taxon sampling since the incorporation of poorly sampled groups, as well as the oversampling of some groups relative to others, may indeed lead to LBA^21^.

## THE ANOMALOUS BEHAVIOR OF ASGARD PROTEINS

Liu et al.^13^ reported that the position of Eukarya was variable in single‐protein trees, branching either within Asgards, as sister group to all Asgards, or as sister group to other Archaea. We previously observed the same phenomenon with the first three published Asgards (Loki 1, 2, and 3) in 36 single‐protein trees^1^, ^5^. The Asgards were also often paraphyletic in these trees, whereas the monophyly of other major archaeal clades was usually recovered (table 1 in Da Cunha et al.^5^). This suggested that the scattered positions of the Asgards were unlikely due to a lack of resolution. Anomalous behavior of Asgard proteins was also reported by Garg et al.^33^ for ribosomal proteins (r‐proteins). These authors suggested that the dispersal of Asgard lineages in r‐protein uTols could reflect mistakes in MAG reconstruction. However, this probably cannot explain every situation. Notably, we observed the same type of anomalous behavior in single‐protein trees with universal proteins encoded by fast‐evolving Archaea of the DPANN superphylum (see fig. S1 in Da Cunha et al.^5^). This raises the possibility that the variable positions of Asgards in single‐protein trees could be due to a fast‐evolving phenotype. Metabolic reconstructions have suggested that Asgards are dependent on symbiotic interactions for both anabolism and catabolism, possibly explaining why they are so difficult to cultivate^34^. It is thus possible that the adaptation of the Asgards to their partners increased the evolutionary rate of some of their proteins.

## THE IMPORTANCE OF THE PROTEIN SAMPLING

**Figure 5:**
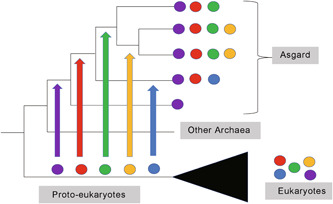
Explaining the patchy distribution of eukaryotic signature proteins (ESPs) in Asgards. Each color circle represents an ESP. Arrows with a similar color indicate their possible transfer from proto‐eukaryotes to Asgard at different periods of Asgard evolution. This is a schematic tree that emphasizes these specific transfers. The present patchy distribution of ESPs probably originated from a more complex pattern involving losses of ESPs in some archaeal lineages and horizontal gene transfer (HGT) between Asgard lineages. HGT of ESPs most likely also occurred between Asgard and other Archaea, as well as from Asgard to proto‐eukaryotes.

Another problem that can bias the topology of the uTol is the composition of the marker data set. Liu et al.^13^ noticed that removing a eukaryotic protein of bacterial origin, the YchF ATPase, from their initial data set of 30 proteins, transformed a 3D tree into a 2D tree. Similarly, we have previously observed that removing the elongation factor EF2 from a data set of 36 proteins transformed a 2D into a 3D tree (fig. 5 in Da Cunha et al.^5^). The fact that a single protein could determine the topology of the uTol based on several dozens of markers highlights the importance of carefully analyzing single‐protein trees before performing the concatenation of their alignments.

The choice of the markers is also critical. We have shown that large proteins tend to support 3D trees, whereas short ones tend to support 2D trees^5^. It is possible that short proteins do not harbor enough informative positions to detect the signal corresponding to the monophyly of Archaea. The data sets used by Liu, Xie, and their colleagues are both enriched in short r‐proteins (80% and 40%, respectively), potentially favoring the 2D topology, and lack several large proteins that gave 3D trees in our previous analysis^6^ (Table 1). In particular, they both lack the large RNA polymerase B subunit. In a recent study of markers conserved between Archaea and Bacteria, Martinez‐Gutierrez and Aylward^21^ have shown that RNA polymerase large subunits have the highest phylogenetic signal (i.e., they more accurately predict a true line of vertical ancestry) and outperformed their full set of 41 conserved archaeal and bacterial markers, despite having a shorter overall alignment length. In contrast, r‐proteins individually tend to carry a low phylogenetic signal. The phylogenetic signal obtained with the concatenation of r‐proteins was much higher than those obtained with single r‐proteins, but remained lower than the signal obtained with the concatenation of the two RNA polymerase large subunits^21^.

**Table 1:** Presence/absence of universal markers giving either 2D or 3D trees.

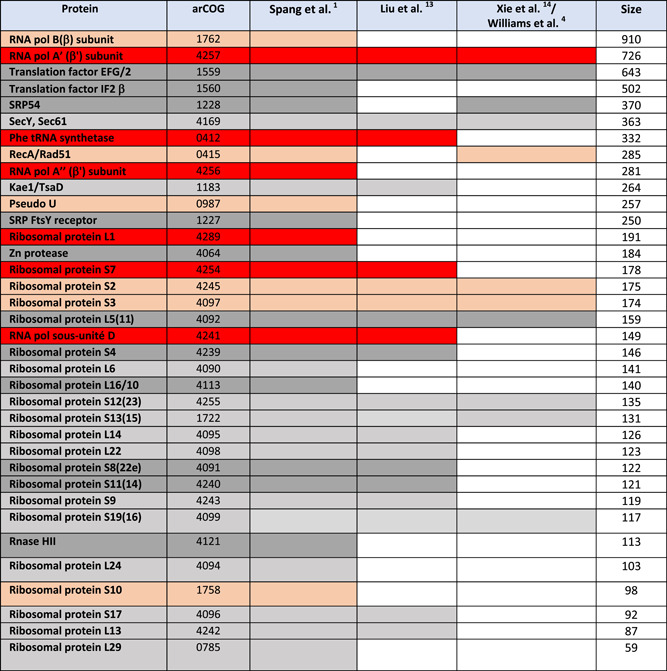

Lists of protein markers used by Spang and colleagues^1^ to construct uTol and reanalyzed by Da Cunha et al.^5^ that were present (colored boxes) or absent (white boxes) in the datasets of, Liu and colleagues^13^ or Xie and colleagues^14^ taken from Williams et al.^4^. In red boxes, proteins giving 3D tree supported by AU test in Da Cunha et al.^5^; in light orange boxes, proteins giving 3D tree not supported by AU test in Da Cunha et al.^5^; in grey boxes, proteins giving 2D tree supported by AU test in Da Cunha et al.^5^; in light grey boxes, proteins giving 2D tree not supported by AU test in Da Cunha et al.^5^. The sizes correspond to the number of amino acids in the trimmed alignment of each marker.

We have previously obtained a robust 3D uTol with the concatenation of the two large RNA polymerase subunits with non‐homogeneous models in the Bayesian framework^5^. Notably, Williams et al.^4^ also obtained a 3D RNA polymerase uTol with our data set and a different non‐homogeneous model (fig. S1 in Williams et al.^4^). They only obtained a 2D uTol after recoding the amino acids of the multiple sequence alignments. Since amino acid recoding strongly reduces the signal in phylogenetic analysis^35^, it is possible that the switch of the RNA polymerase uTol from a 3D to a 2D tree after recoding is due to a lower signal supporting the monophyly of Archaea.

It has been suggested that 3D trees could be due to the attraction of Eukarya by Bacteria (Figure 3)^4^. However, the inclusion of the three eukaryotic RNA polymerases in the RNA polymerase uTol, to reduce the length of the eukaryotic branch, did not produce a 2D uTol^28^, suggesting that the 3D RNA polymerase uTol is not biased by an attraction between Eukarya and Bacteria (Figure 4).

More globally, an important step in phylogenetic reconstructions is the filtering of ambiguous sites through various tools to reduce the noise that can be generated when aligning universal markers, especially with large data sets. This step can also deeply affect the final phylogenetic tree^23^, ^36^ and considering the difficulty to find an appropriate balance between signal and noise filtering, it is possible that excessive trimming algorithms would tend to lead toward 2D uTols by decreasing an already relatively low archaeal‐specific signal carried within short protein sequences.

## HGT BETWEEN ASGARDS AND PROTO‐EUKARYOTES COULD EXPLAIN THE PATCHY DISTRIBUTION OF SOME ESP_S_

The characterization of new Asgard lineages led to the identification of several new ESPs^13^, ^14^. Notably, both teams noticed that many ESPs are lineage‐specific and that many were missing in the Asgard lineages that are sister groups to Eukarya in their analyses. This patchy distribution has already been observed^37^, ^38^, but is even more visible with the ongoing expansion of the new Asgard lineages. Intriguingly, despite this major expansion, several ESPs are still only present in a few Asgard lineage^13^, ^14^. This is for instance the case of tubulin, which is only present in the Odin lineage^2^, ^14^.

In both 2D and 3D models, the extremely patchy phyletic distribution of ESPs requires to involve many losses and/or transfers between archaeal lineages. In the 2D scenario, one should further assume that Eukarya emerged from an extinct Asgard lineage that acquired the whole set of ESPs now distributed among the various Asgard lineages^37^. In both models, it is especially difficult to explain the existence of ESPs presently restricted to a single or few Asgard lineages.

An alternative hypothesis that could more easily explain the patchy distribution of Asgard ESPs is that some ESPs were recruited by Asgards from proto‐eukaryotes (i.e., members of the eukaryotic lineages that predated the last eukaryotic common ancestor [LECA]). Such horizontal gene transfer (HGTs) might have occurred either early in Asgard evolution, with the corresponding ESPs being present in most or all Asgards, or later, during the diversification of Asgard lineages, explaining the restricted distribution of the corresponding ESPs (Figure 5). Notably, such proto‐eukaryotic HGT hypothesis has been previously proposed to explain the presence of ESPs in a few Bacteria, such as actin or tubulin, very similar to the eukaryotic ones but branching as a sister group to Eukarya^39^, ^40^, ^41^, ^42^.

Importantly, this proto‐eukaryotic HGT hypothesis would explain why some Asgard ESPs are much more similar to their eukaryotic homologs than to archaeal ones. This is the case of Odin tubulin, which is much more closely related to eukaryotic tubulins than to the tubulin found in Thaumarchaeota (Extended Data fig. 6 in Zaremba‐Niedzwiedzka^2^). Similarly, most Asgard actins are much more similar to eukaryotic actins and actin‐related proteins (ARPs) than to archaeal crenactins^43^, ^44^. In the case of actin, the proto‐eukaryotic HGT hypothesis is strongly supported by phylogenetic analyses showing that the various forms of actins present in Asgards branch between the various clades of eukaryotic actin paralogs (cytoplasmic actin and ARPs) that were already present in LECA^43^, ^44^. Also, corroborating this hypothesis, Nasir et al.^7^ concluded from a comparative analysis of conserved folds that ESPs are of relatively recent origin and could correspond to late HGTs between Archaea and Eukarya.

**Figure 6:**
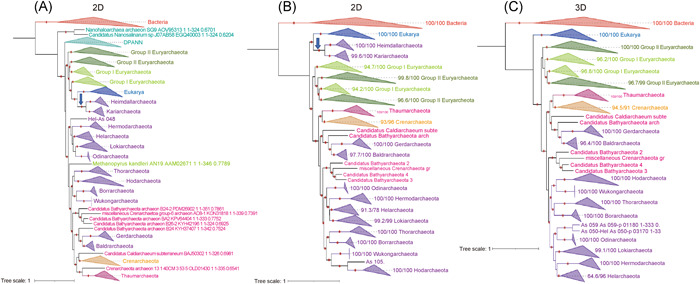
Phylogenetic trees of the universal protein Kae1/TsaD highlight the existence of different versions of Asgard proteins. From left to right. Initial 2D tree with DPANN at the base of Archaea and the fast‐evolving euryarchaeon *Methanopyrus kandleri* branching within paraphyletic Asgard (A), the 2D tree after removing fast‐evolving species (DPANN, *M. kandleri*) with Asgard divided into two monophyletic groups, one (two groups) sister group to Eukarya and the other (10 groups) branching between Euryarchaeota and the TACK clade (B) and the 3D tree obtained after further removing the two Asgard clades sister group to Eukarya (C). We observed in red several MAGs annotated as putative Bathyarchaota or Aigarchaeota that could be results of HGT or new groups of Asgard (C). The trimmed alignment provided of the Kae1/TsaD protein was selected from https://doi.org/10.5281/zenodo.4624280 ^13^. The maximum likelihood trees were constructed using IQ‐TREE version 1.6.1239 under the model of evolution according to the MFP option for model selection. Values at nodes represent branch supports calculated with the Shimodaira–Hasegawa‐like approximate likelihood ratio test (aLRT) (10,000 replicates) and UFBoot (10,000 replicates), and a dot is indicated if the aLRT >80 or UFBoot >95. The scale bar represents the average number of substitutions per site. The trees will be available on figshare (https://figshare.com/s/9e93072a77c3e014efd0).

Notably, the proto‐eukaryotic HGT hypothesis is relevant in both the 2D and 3D scenarios. This implies that, independently of the correct scenario, the study of ESPs could provide important information about eukaryogenesis by identifying intermediate steps in the evolution of these proteins. Moreover, this hypothesis implies that ancient Asgards were already diversified before LECA and shared their biotopes with proto‐eukaryotes. Consequently, studying the environmental distribution of modern Asgards could provide critical insights into the nature of the biotopes in which proto‐eukaryotes were thriving.

In the modern biosphere, HGTs between Eukarya and Bacteria are especially prevalent between species living in close association. For instance, some intracellular Bacteria have acquired many eukaryotic proteins from their hosts^45^. Symbiotic associations between Archaea and Eukarya have been also described, such as methanogens thriving in protists from different eukaryotic lineages^46^ or *Cenarchaeum symbiosum* inhabiting in marine sponges^47^. It is thus possible that some Asgard ancestors were symbionts of proto‐eukaryotes, exchanging genes with their hosts. Interestingly, the first cultivated Asgard, *Candidatus* Prometheoarchaeum syntrophicum, is a symbiotic organism living in association with a methanogen and a bacterium^34^. It could be worth exploring if some Asgards are living in symbiosis with modern Eukarya by looking for Asgard signatures in various types of eukaryotic cells.

## HGT BETWEEN ASGARDS AND PROTO‐EUKARYOTES COULD ALSO EXPLAIN THE ODD POSITION OF SOME ASGARD LINEAGES IN SINGLE PROTEIN TREES

If HGTs took place between proto‐eukaryotes and Asgards, one can wonder if they also involve universal markers. Examining the single‐protein trees obtained by Liu and colleagues, we found a possible illustration of such a scenario in their uTol of the Kae1/TsaD protein (Figure 6A). Kae1/TsaD catalyzes the synthesis of threonylcarbamoyladenosine (t6A), a universal modification of transfer RNAs^48^. We previously obtained a 3D phylogeny for this protein before the discovery of Asgards^49^, whereas Liu et al.^13^ obtained a 2D tree. In this tree, Asgards are paraphyletic with the Heimdall and Karia lineages branching as sister groups to Eukarya, whereas other Asgards form three distinct clades branching within Archaea (Figure 6A). This suggests that the latter correspond to the ancestral archaeal version of Kae1/TsaD that was replaced by a proto‐eukaryotic‐like version in a common ancestor to the Heimdall and Karia lineages (blue arrow). We also noticed that Archaea were rooted within DPANN and that *M. kandleri* did not branch with other Euryarchaeota (in green), testifying again for the difficulty to correctly position fast‐evolving species. When we removed these species from the data set, the 2D tree then obtained was essentially a 3D tree with Heimdall and Karia, still branching with Eukarya, and the rest of Asgards being nested within monophyletic Archaea (Figure 6B). Predictably, removing the Heimdall and Karia lineages from the uTol produced a 3D tree (Figure 6C) instead of a 2D tree.

Surprisingly, MAGs of Bathyarchaea (in red) did not form a robust clade with Thaumarchaeota in the Kae1/TsaD trees, as in most archaeal phylogenies^13^, but were interspersed with Asgards (Figure 6). This could reflect mistakes in MAG annotation or HGTs between Asgards and Bathyarchaea, which co‐occur in various environments^11^.

We suspect that the HGT hypothesis could also explain our previous observation of the strong impact of EF2 from one Asgard species of the Hodar lineage (formerly Loki 3) on the uTol^5^. This would explain why Hodar EF2 contained specific eukaryotic‐like insertions that were missing in all other Asgards^5,50^. Remarkably, these EF2 branched as sister group to various eukaryal EF2 paralogs in phylogenetic analyses, whereas all other Asgards EF2 branched as a monophyletic group within Archaea (fig. 2 in Narrowe et al.^50^ [Correction added on June 6, 2022, after first online publication: The citation, ‘Hecker et al.^49^’ has been amended to ‘Narrowe et al.^50^’ in this sentence.] see also fig. 1 in Da Cunhua et al.^5^), reminiscent of the situation previously described for Kae1/TsaD. This suggests that the archaeal version of EF2 was replaced in a common ancestor of the Hodar lineage by a proto‐eukaryotic version.

The identification in specific Asgard lineages of universal proteins that are much more similar to their eukaryotic homologs than to their homologs in all other Asgard lineages indicates that HGTs between proto‐eukaryotes and Asgard could be another confounding factor favoring 2D over 3D uTols.

## CONCLUSION

Many recent observations about Asgards have been systematically interpreted in the 2D scenario framework. For instance, Avci et al.^51^ suggested that condensed nucleoid observed in some Asgard cells could correspond to transition steps in the formation of the nucleus of eukaryotic cells, whereas Rambo et al.^52^ concluded that Asgard *Caudoviricetes* have both archaeovirus and eukaryovirus characteristics because they encode informational proteins with homologs in eukaryotic viruses of the phylum *Nucleocytoviricota.* However, some Bacteria also have condensed nucleoid^53^ and homologs of proteins encoded by *Nucleocytoviricota* are also present in bacterial *Caudoviricetes* ^54^, ^55^, ^56^. Moreover, the recent characterization of Asgard viruses revealed three groups of viruses typical of other Archaea, including two groups (*Caudoviricetes* and *Tectiliviricetes*) that are present in Bacteria but not in Eukarya^57^, ^58^. Asgard cells are rather small, with typical archaeal lipids, hence do not exhibit obvious intracellular complexity and probably lack phagocytosis ability^59^. The discovery of Asgards thus did not reduce the formidable divide existing between the archaeal and eukaryotic phenotypes. The transformation of an archaeon into a eukaryote still involves many transitions that have never been observed in nature^60^, ^61^, ^62^. As pointed out by Nasir et al.^7^, all known organisms forming intimate endosymbiotic or ectosymbiotic associations have maintained their domain identity. They noticed that “even mitochondria are still recognizable as highly‐reduced Bacteria despite billions of years of coexistence inside the eukaryotic cell.”

Despite the apparent congruence between 2D phylogenies displaying Eukarya branching within the Asgards and the abundance of ESPs in these latter, we argue that the debate between the 2D and 3D uToLs is not closed. We have here described several factors that could artifactually favor the 2D over the 3D topology in uTol analyses and hypothesized the existence of HGTs between Archaea and proto‐eukaryotes that could easily explain the presence of some ESPs in the Asgards. Taking all these observations and hypotheses into consideration, Box 1 summarizes our advices to improve future phylogenetic analyses and finally fulfill Darwin's dream to reconstruct the tree of life.

Box 1

 

- Use a similar number of species for each domain and for each phylum within the domain based on more recent domain‐specific phylogenies. Avoid overrepresentation of some groups.
- Do not include fast‐evolving species, such as *M. kandleri*, Korarchaeota, or DPANN in Archaea or candidate phylum radiation in Bacteria. The position of these species in their respective domain should be tested independently of the uTol.
- Search for new universal proteins from the literature. Some of them are removed from automatic program searches because of paralogy or because they are split into two or more proteins in some groups. This probably explains why some data sets do not include the B subunit of RNA polymerase.
- Examine single‐protein trees to detect paralogy and HGT between and within domains based on previous knowledge of domain phylogeny. Remove species affected by HGT and fast‐evolving paralogous groups.
- Search markers with the best phylogenetic signal and congruence using tools such as the Tree Certainty metric^21^. Best markers should recover the monophyly of well‐established clades within domains.
- For concatenation, test different combinations of subsets of proteins to detect markers that could bias the signal.
- Test different trimming with different sets of species/markers and verify the absence of domain inversion/substitution mismatches. Apply different models for tree reconstruction.

## Supporting information

Supporting information.
