# Supplementary material for: The expanding Asgard archaea and their elusive relationships with Eukarya
Source: mLife. 2022 Mar 24;1(1):3–12. doi: 10.1002/mlf2.12012 (PMC10989751; doi:10.1002/mlf2.12012)
Supplement: Supplementary file 1 — Supporting information. [file MLF2-1-3-s001.docx]

| **Proteins** | **arCOG** | **Spang** | **Liu** | **Xie/Williams** | **Size** |
| --- | --- | --- | --- | --- | --- |
| **RNA pol B(β) subunit** | 1762 |  |  |  | 910 |
| **RNA pol A’ (β') subunit** | 4257 |  |  |  | 726 |
| **Translation factor EFG/2** | 1559 |  |  |  | 643 |
| **Translation factor IF2 β** | 1560 |  |  |  | 502 |
| **SRP54** | 1228 |  |  |  | 370 |
| **SecY, Sec61** | 4169 |  |  |  | 363 |
| **Phe tRNA synthetase** | 0412 |  |  |  | 332 |
| **RecA/Rad51** | 0415 |  |  |  | 285 |
| **RNA pol A’’ (β') subunit** | 4256 |  |  |  | 281 |
| **Kae1/TsaD** | 1183 |  |  |  | 264 |
| **Pseudo U** | 0987 |  |  |  | 257 |
| **SRP FtsY receptor** | 1227 |  |  |  | 250 |
| **Ribosomal protein L1** | 4289 |  |  |  | 191 |
| **Zn protease** | 4064 |  |  |  | 184 |
| **Ribosomal protein S7** | 4254 |  |  |  | 178 |
| **Ribosomal protein S2** | 4245 |  |  |  | 175 |
| **Ribosomal protein S3** | 4097 |  |  |  | 174 |
| **Ribosomal protein L5(11** | 4092 |  |  |  | 159 |
| **RNA pol sous-unité D** | 4241 |  |  |  | 149 |
| **Ribosomal protein S4** | 4239 |  |  |  | 146 |
| **Ribosomal protein L6** | 4090 |  |  |  | 141 |
| **Ribosomal protein L16/10** | 4113 |  |  |  | 140 |
| **Ribosomal protein S12(23** | 4255 |  |  |  | 135 |
| **Ribosomal protein S13(15** | 1722 |  |  |  | 131 |
| **Ribosomal protein L14** | 4095 |  |  |  | 126 |
| **Ribosomal protein L22** | 4098 |  |  |  | 123 |
| **Ribosomal protein S8(22e** | 4091 |  |  |  | 122 |
| **Ribosomal protein S11(14** | 4240 |  |  |  | 121 |
| **Ribosomal protein S9** | 4243 |  |  |  | 119 |
| **Ribosomal protein S19(16)** | 4099 |  |  |  | 117 |
| **Rnase HII** | 4121 |  |  |  | 113 |
| **Ribosomal protein L24** | 4094 |  |  |  | 103 |
| **Ribosomal protein S10** | 1758 |  |  |  | 98 |
| **Ribosomal protein S17** | 4096 |  |  |  | 92 |
| **Ribosomal protein L13** | 4242 |  |  |  | 87 |
| **Ribosomal protein L29** | 0785 |  |  |  | 59 |

Table S1: Lists of protein markers used by Spang and colleagues [1] to construct uTol and reanalyzed by Da Cunha et al [5] that were present in the datasets of, Liu and colleagues [11] or Xie and colleagues [12] taken from Williams et al. [4]. In red boxes, proteins giving 3D tree supported by AU test in Da Cunha et al. [5], in light orange boxes, proteins giving 3D tree not supported by AU test in Da Cunha et al [5]. In grey boxes, proteins giving 2D tree supported by AU test in Da Cunha et al. [5]. In light grey boxes, proteins giving 2D tree not supported by AU test in Da Cunha et al. White boxes indicate the absence of these proteins in the dataset.

The sizes correspond to the number of amino-acids in the trimmed alignment of each marker.
